# New horizons in advance care planning: narratives, identity and cultural humility

**DOI:** 10.1093/ageing/afag191

**Published:** 2026-07-01

**Authors:** Sarah A Hopkins, Louisa Polak, Sarah E Hoare, Simon N Etkind, Luke Stalley, Roberta Lovick, Isla Kuhn, Rhian Simpson, Rowan H Harwood, Stephen Barclay, Michael Kelly

**Affiliations:** Department of Public Health and Primary Care, University of Cambridge, Cambridge, UK; Department of Public Health and Primary Care, University of Cambridge, Cambridge, UK; Department of Public Health and Primary Care, University of Cambridge, Cambridge, UK; Department of Public Health and Primary Care, University of Cambridge, Cambridge, UK; Cambridge University Hospitals NHS Foundation Trust, Cambridge, UK; Department of Sociology, University of Zurich, Zürich, ZH, Switzerland; Patient representative, Great Yarmouth, UK; Cambridge University Medical Library, School of Clinical Medicine, University of Cambridge, Cambridge, UK; Department of Public Health and Primary Care, University of Cambridge, Cambridge, UK; School of Health Sciences, Queens Medical Centre, University of Nottingham, Nottingham, UK; Department of Public Health and Primary Care, University of Cambridge, Cambridge, UK; Department of Public Health and Primary Care, University of Cambridge, Cambridge, UK; Cambridge Public Health, University of Cambridge, Cambridge, UK

**Keywords:** advance care planning, ACP, narrative, identity, culture, older people

## Abstract

Advance care planning (ACP) is considered important for patient-centred care in serious illness, yet evidence it benefits patients is inconsistent. This reflects a lack of clarity over how ACP interventions are supposed to achieve their intended goals and what these goals should be, leading to variation in implementation. This article seeks to address this knowledge gap by focusing on the role of narratives.

Although ACP draws on patients’ illness narratives (their personal stories of illness, including hopes and fears), the role of these narratives is often unacknowledged. We systematically reviewed the evidence on narratives and ACP and synthesised the findings with broader multidisciplinary literature.

Attending to patient narratives can be a route to achieving many goals of ACP (including preserving patient autonomy, strengthening relationships and preparing for end-of-life) and can help patients to maintain their identity, which is important for well-being but is disrupted by illness and frailty. The values underpinning ACP are culturally-specific, however, with less relevance for some patients: better appreciation of this is a step towards cultural humility and more equitable care. The role of clinicians in contributing to patient life narratives through ACP is controversial. Some argue that clinicians should shape their patient’s narrative, helping them to make meaning at the end-of-life, while others have problematised expectations that patients tell, and clinicians facilitate, ‘good death’ stories.

Synthesising existing knowledge provides clinicians with insights on how to approach end-of-life conversations through framing discussions around patient narratives and lays the foundations for future research exploring narrative approaches to ACP.

## Key points

- Attending to patient narratives (personal stories of illness) is an under-recognised route to achieving many goals of ACP.
- When ACP attends to a patient’s narrative, it can help that person to maintain their identity—important for well-being.
- Appreciating the cultural specificity of the narratives underpinning ACP is a step towards cultural humility and equitable care.
- The extent to which clinicians should, and do, shape patient life narratives through ACP is contentious and under-researched.
- A better understanding of the role of narratives in ACP can help clinicians to reflect on and refine their own practice.

## Introduction

A patient’s narrative about their illness—their personal story of their illness, their experiences, feelings, fears, hopes and expectations for the future—is inherently intertwined with the plans they make for their future, including their future care. Serious illness, including frailty, can disrupt the narratives an individual had previously relied on to make sense of their life [1, 2]. To make sense of their new situation, they must reshape their stories [3]. Helping patients to make sense of their story is recognised as clinically important [4] and has been described as the ‘moral core of doctoring’ [5]. Narrative medicine, which emphasises the importance of attending to patients’ stories, has consequently gained traction over recent decades [6].

Elements of a narrative approach are present within many models of advance care planning (ACP). For example, several ACP training programmes, including the internationally-used Serious Illness Care Programme, implicitly attend to patient narratives; [7] helping patients to reshape their narrative was a tacit aim of recent recommendations for serious illness conversations [8]. Narratives, however, are often invisible in the process of ACP: their role is often unacknowledged. Consequently, whilst narratives are intrinsically entwined with ACP, there is a knowledge gap about how they contribute to its goals and their impact on its outcomes; existing research is scattered and variable in quality. Here we unpack this black box by reviewing and synthesising the available evidence.

A better understanding of the role of narratives in ACP is timely, given that ACP is an important aspect of patient-centred care in the context of serious illness and an international policy priority [9–11], yet evidence that it benefits patients is inconsistent [11–13]. For example, while a 2022 systematic review of randomised controlled trials (RCTs) found that ACP increased the likelihood of patients receiving end-of-life care consistent with their preferences in only 3 out of 12 studies [13], a 2025 meta-review of reviews found improvement in this domain in 15 out of 17 studies [14], and a review looking specifically at ACP for people living with dementia found improvements in 3 out of 3 included studies [15]. Evidence also varies for other outcomes, including quality of life (with 5 reviews and 14 RCTs finding no effect, but 8 reviews finding improvement), depression and place of death [13–15]. One reason for this mixed efficacy is heterogeneity in study interventions [13, 16], which itself reflects a lack of clarity over how ACP interventions are supposed to achieve their intended goals [17], and what these goals should be [18]. A second reason is that ACP, and broader concepts of a ‘good death’, are based on tacit culturally-situated values that are unlikely to be relevant for all patients [19, 20]. For example, ACP prioritises autonomy and ‘may not speak to communities shaped by family decision making, shared responsibility, and socio-spiritual values emphasising collectivism, such as among some South Asian, African, and Caribbean communities’ [21].

Identifying and unpacking the role of narratives in ACP may help improve patient outcomes by increasing our understanding of how ACP works, its goals and its underpinning cultural values. This review aims to provide a new perspective on these areas, exploring whether models drawing on narrative medicine, with its focus on helping patients make sense of their experiences, may be useful in pursuit of a range of patient-centred ACP goals. This is of particular relevance to older people living with frailty and multimorbidity because research has found that these individuals can struggle to make sense of what is happening to them [22], and this is associated with reduced wellbeing [23].

We consider links between narratives and ACP in relation to (i) achieving the goals of ACP, (ii) improving patient outcomes and (iii) the culturally-specific assumptions that underpin ACP. Finally, (iv) we explore the role of clinicians in contributing to patient narratives through ACP.

Box 1 Definitions.

**Advance care planning (ACP)**: ‘ACP is a voluntary process of person-centred discussion between an individual and their care providers about their preferences and priorities for their future care, while they have the mental capacity for meaningful conversation about these. The process, which is likely to involve a number of conversations over time, must have due consideration and respect for the person’s wishes and emotions. As a result, the person should experience a greater sense of involvement and the opportunity to reflect and share what matters most to them’ [10].

**Narrative**: ‘An account of a series of events, facts, etc., given in order and with the establishing of connections between them; a narration, a story, an account’. [24] The Oxford English Dictionary defines narrative in several ways, reflecting the term’s use in different disciplines. Narratives can operate on several levels: this review focuses on ‘personal narratives’—the stories individuals tell themselves and others—and ‘cultural narratives’—the narratives that shape and are shaped by how a society or culture thinks about and represents itself [25–27].

**Life narrative** or **life story**: The evolving explanations individuals give to themselves and others, about their perceived past, present and imagined future. It is proposed that our sense of self—our notion of who and what we are—comes from the life stories we tell [28, 29, 30]. Although based on biographical facts, life narratives are also curated—in telling them, we select elements that we consider salient, given our individual and societal values and preconceptions, and to whom we are telling them [31, 32].

**Narrative medicine:** medicine practised with ‘the ability to acknowledge, absorb, interpret, and act on the stories and plights of others’ [6]. Within this review, we use the term ‘attending to patient narratives’ to refer to practising in this way. Narrative medicine emphasises that meaning is derived from the stories that we tell, and that listening, marshalling and interpreting these stories are important components of patient care [33].

Box 2 Methods and search results.

Research on narratives and ACP was systematically searched, reviewed and analysed thematically. These systematic review findings were then synthesised with relevant background literature, drawing on both the interdisciplinary literature on narratives and the broader ACP literature (please see Supplementary Appendix S1 for more details on the review method). The search returned 311 unique articles, of which 19 met inclusion criteria and a further 7 were identified from reference searching (please see Supplementary Appendix S2 for PRISMA flow diagram) [34].

The review was inclusive of a range of evidence types, including quantitative and qualitative empirical evidence, bioethical and philosophical essays, review articles and commentaries. Descriptions of a one-off personal experience as a patient or clinician (personal narratives) were not included. Our broad approach to evidence types was taken because this is an area where both empirical data and underpinning theory are important: identifying putative underpinning mechanisms is a step in the development of robust complex interventions, allowing these mechanisms to be tested empirically [35]. The range of evidence types meant that comparing the quality of the evidence between included papers was not meaningful, instead the nature of the evidence supporting each finding is reported.

Two authors (S.A.H., L.P.) identified the main themes from the articles identified in the systematic review, which fell into four categories: (i) how patient narratives help achieve the goals of ACP, (ii) the impact on patient outcomes, (iii) the culturally-specific assumptions that underpin ACP and (iv) the role of clinicians in contributing to patient narratives through ACP. They then looked at each paper in more detail and extracted findings relevant to narratives and ACP within these four categories. Next, and with input from the multidisciplinary authorship team, they synthesised these themes with reference to background literature from two main areas. First, the philosophical, psychological and sociological literature on narratives, and the literature on narrative medicine. This is large and is important because of the central role of narratives in patients’ experience of illness and clinician-patient interactions [1, 5, 6] but some has never previously been applied to ACP. Second, the broader literature on ACP: empirical evidence, review articles, guidelines and policy documents were used as examples to work with, as well as to situate and contextualise the findings.

ACP is a field laden with values, where the meaning of ‘good’ is often culturally situated as, e.g. in a ‘good death’. These values are sometimes tacit, taken for granted in research and debate about ACP, and they limit the transferability of research findings to different sociocultural settings. In this review, the authors have tried to identify these value judgements, so that they can be considered by clinicians, researchers and policy makers and discussed openly with patients and the public.

## How patient narratives help achieve the goals of ACP

Clinicians attending to patient narratives is a route to achieving many goals of ACP, including (a) preserving patient autonomy, (b) strengthening relationships and (c) preparing for the end of life (Table 1) [18]. This is important because the existing lack of clarity over *how* ACP achieves its goals is one reason for the wide variation in outcomes from ACP trials [17]. Without this understanding, interventions may focus on aspects of ACP that do not lead to benefits for patients [36]. An example of this is the presence of a plan in a patient’s notes, which is often used as an outcome measure in ACP trials but is not consistently associated with patient benefit [8, 13]. A better understanding of how ACP achieves its goals may improve the efficacy and effectiveness of ACP interventions by ensuring key aspects are not lost in implementation [35].

**Table 1:** How patient narratives help achieve the goals of ACP.

ACP goal	How patient narratives help achieve this goal
**1. Promoting patient autonomy:** **a) identifying values** Focusing on patient narratives during ACP can give both patient and clinician insight into the patient’s values, goals and preferences [41–44].	An interdisciplinary conceptual analysis proposes that ACP should begin with attending to a patient’s identity as revealed through their narrative, and from this their values can be ‘discovered’, and then used as the basis for patient and clinician to co-create concrete goals, and finally to make decisions [41]. A qualitative interview study with patients and clinicians concluded that ‘deciphering people’s stories gives us insight into their values, the mental constructs that drive their decision making, and the goals that they have for their own health care’ [45]. In a bioethical essay, Jacquemin argues it is only through listening to an individual’s life narrative that future decisions can be made in keeping with that person’s identity [44].
**1. Promoting patient autonomy:** **b) feeling a sense of control** ACP conversations may help patients to feel a sense of autonomy, or control, by assisting them to tell a narrative in which they are in control of events (a so-called agency narrative) [10, 28, 46].	ACP encourages patients to construct a narrative about how their choices for their future healthcare are the right thing to do because they align with their values (rather than because they have no other option). For example, ‘I have chosen not to return to hospital because for me quality of life is more important than quantity’. This is important because qualitative data show that not all decisions involve agency narratives e.g. ‘I had no choice but to… take statins, the doctor told me I had to’ [47]. By creating the space to express preferences through ACP, and by then validating the patient’s choice, clinicians may help strengthen agency narratives that place the patient in control.
**1. Promoting patient autonomy:** **c) identifying values with individuals who lack legal decision-making capacity for ACP** Focusing on patient narratives during ACP can help identify the values of individuals who lack legal decision-making capacity [48, 49].	For individuals who lack legal decision-making capacity for ACP, their narratives might point to their goals and values and form a basis on which they can contribute to decisions about their future care, as outlined in bioethical essays focused on dementia [48] and intellectual disability [49]. ACP can also help with a patient’s legacy by ensuring the narratives others tell about an individual are consistent with the values that individual espoused in earlier parts of their life. Rather than emphasising the importance of the individual’s life narrative, here the focus is on stories told about that individual after they die by those around them, as proposed in a philosophically-orientated bioethical essay [50].
**2. Strengthening relationships** The process of listening to patient narratives during ACP may help to strengthen therapeutic relationships [51, 52].	As suggested more broadly by proponents of narrative medicine [6], and argued specifically in the context of palliative care [51, 52], listening to narratives builds trust between provider and patient, and shows respect to the storyteller. Narratives may increase shared understanding of the meaning of pain and illness, relationships and religion, which might be particularly important when provider and patient are from different sociocultural backgrounds [42, 51, 52]. Some authors suggest that listening to patient narratives can help to address existing disparities in end-of-life care between Black and White patients [43, 51].
**3. Preparing for end-of-life:** **a) making sense of illness** The process of telling their story during ACP can help patients to make sense of their illness and find meaning in life [44, 51–53].	For the patient, telling their story and making decisions may help them to make sense of their illness [51–53], assess their own life journey and find meaning in life [44, 54]. For example, this process might help patients to re-prioritise and work out ‘what really matters’ in life, allowing them to feel they are making the most of the time they have left [55]. These benefits of sharing stories through ACP are supported by a small mixed methods study of a narrative ACP intervention [43]. This sense- and meaning-making process is sometimes framed as preparing for the end of life [18].
**3. Preparing for end-of-life:** **b) giving death meaning** ACP conversations may help patients to tell a redemption narrative, finding meaning in nearing death [8, 51, 52].	ACP conversations can also help patients to present narratives where death itself is accepted and possibly also given value or meaning [8, 51, 52], e.g. helping patients to ‘not only endure pain and suffering but find comfort and make meaning of this experience’ [52]. By encouraging patients to use approaching death as an opportunity to identify what matters to them, clinicians can help patients tell a story of finding redemption in adversity: of being someone who can and will make the best of a bad situation. This so-called ‘redemption narrative’ [28, 56] is an example of positive reframing (focusing on ways the stressor might be positive or beneficial), which is cited as an explicit objective of palliative care [8].
**‍4. Maintaining identity** Sharing narratives and making decisions through ACP can help individuals to maintain their identity ‘in the present’ [41, 52, 54, 57].	By helping an individual to make sense of their life and illness, ACP contributes to an individual’s current life story and thus their current sense of self and identity [54]. Through incorporating plans for the future and end-of-life preferences into this life story, ACP allows the future to become part of the individual’s present identity [57].

As well as contributing to achieving these accepted ACP goals, when ACP attends to a patient’s narrative, it can help that person to make sense of their story—sometimes described as the ‘moral core of doctoring’ [4]—and maintain their sense of self and identity (Table 1, goal 4). These outcomes have not been much emphasised in the ACP literature to date [18], but may be valuable to patients: maintaining a sense of self is widely considered to be important for well-being [37], but it can be disrupted by illness and frailty [1, 23], and its loss is considered a central component of suffering [38]. Consequently, clinicians might do well to first focus on patient’s narratives—and to see their role in sense-making and identity as important in its own right—before making a plan.

This is especially important because ACP conversations may *inadvertently* contribute to a patient’s sense of self by affecting their life story, even if this is not the clinician’s intention. Given empirical evidence from the field of narrative psychology showing association between aspects of narrative identity and well-being [28, 56, 39, 40], it is helpful for clinicians to be aware of this, so they can choose to attend to these outcomes more consciously.

Recognising the role of ACP in sense-making and identity work highlights potential synergy between ACP and other narrative-centred interventions. Sharing stories through ACP can be conceptualised as part of life review—which includes focusing on life history, attainments and social roles—and which meta-analyses have found to be associated with improved quality of life for some patient populations, including older adults [58, 59]. One qualitative study with palliative patients found that sharing narratives focused on personal memories, without explicitly discussing priorities for future care, helped individuals to maintain their sense of self [60]. This may be helpful for those who do not wish to make plans for the future, but still struggle to maintain their sense of self, as can occur for individuals living with frailty [23]. Such populations may be underserved by palliative care approaches that emphasise the importance of making a plan, without attending to underlying narratives. Whilst arguments for the benefits of narrative approaches in older age have been made previously [61, 62], to our knowledge, the idea of combining these approaches with ACP is novel.

## Improving patient outcomes by using narratives during ACP

While the previous section makes evident the theoretical benefits of attending to patient narratives during ACP, the impact on clinical practice and patient care is yet to be properly explored. We found only one small interventional study addressing whether attending to narratives as part of ACP changes patient or relative outcomes. This was a single site pre/post study of an education intervention providing training in the 3-Act Model, a narrative approach to goals of care conversations. This approach involves first listening to a patient’s story (‘What would you like us to know about you as a person?’) to understand their values, then sharing medical information and finally making shared decisions [63, 64]. Doing so increased rates of goals of care documentation completion and consults by palliative care, although no other patient outcomes were measured [65]. Whilst there has been no empirical research into the effect of attending to patient narratives during ACP on patient quality of life, experience of care or on outcomes for bereaved relatives, a qualitative observational study looking at ACP with older adults in their own homes in Norway found that stories were a way that patients asserted their identity with their clinicians, as well as a means to express values implicitly and to establish relationships [66]. More research is needed to explore this in different settings and cultural contexts.

More broadly, several studies have looked at narrative medicine ACP education interventions for healthcare professionals and found that these improved measures of provider competence and confidence [63–65, 67–70]. One small study looked at patient readiness for ACP before and after a narrative medicine reflective writing session and found increased ACP readiness [71]. A 2019 scoping review of narrative interventions in palliative care concluded that there was a ‘striking dearth’ of studies, and that ACP was a tractable area for future research [67].

Looking at the narrative strategies people use when speaking about their preferences for the end of life may help clinicians to initiate and navigate difficult ACP conversations. We found only one empirical study in this area: a US qualitative focus group study looked at the narrative strategies used by patients when discussing ACP [72]. They found that narrating stories of others’ end of life care and hypothesising about what they would tell loved ones about their wishes were common narrative strategies and allowed the speaker to simultaneously engage with and distance themselves from difficult topics. Further research is needed to gain a better understanding of the diverse narrative strategies used in different cultures, diseases and life stages and how this can be used to improve clinical practice.

## Culturally-specific assumptions that underpin ACP

We now turn to the shared assumptions and values that have informed ACP and shaped its goals. The culturally-specific nature of these assumptions and values, or ‘cultural narratives’, means they have less relevance for some patients than for others, something under-recognised in the general ACP literature [21]. This may contribute to the fact that people from minoritised groups often experience worse care at the end of life than people who are White, English speaking and socially advantaged [21]. More broadly, the differences between communities’ shared narratives limit the transferability of knowledge about ACP—what works well within one community may not work in another.

The dominant cultural narratives underpinning ACP centre on (1) control (agency narratives) and (2) acceptance through finding meaning in adversity (redemption narratives), as demonstrated by national and international policies and guidance [9, 10, 73, 74]. The importance of control can be seen in the UK’s Universal Principles for Advance Care Planning guidance, which states ‘Many people feel more confident that they have gained more control of their own lives through doing this’ [10]. With respect to finding meaning in adversity, the Lancet Commission’s Report emphasises the value of death: ‘Philosophers and theologians from around the globe have recognised the value death holds for human life’ [73]. This reflects broader Western cultural ideals of a ‘good death’—the importance of control and acceptance have been illuminated by policy analysis and integrative reviews [19, 20, 75]—and is in keeping with the high value often attributed to autonomy in Western medical ethics [76].

We found that the cultural narratives underpinning ACP are rarely explicitly acknowledged, and instead constructs like individual autonomy and peaceful acceptance of death are often taken for granted as inherently good. Evidence from the field of narrative psychology, conducted mainly in North America, has found agency and redemption narratives to be associated with better well-being and mental health [56, 39, 40] although whether this relationship is causal is not known [28], and this systematic review found no research on this in the context of ACP.

### The need for cultural humility in ACP

Some authors have argued that ACP is a way of helping patients to tell personal narratives that align with the cultural narrative of control, and that this has less relevance in some cultures [77, 78]. Drawing on surveys and qualitative interviews, Gordon and Paci suggested control narratives had less salience for some patients and clinicians in Italy, where relational autonomy was more dominant and the good life was spoken of in terms of tranquillity and serenity rather than self-determination [78]. Writing in a book about ethical challenges in multi-cultural care, Searight highlighted that suffering is considered to have meaning in some cultures, and making an advance care plan to limit treatment may demonstrate a loss of faith [77]. Moreover, some cultures consider that saying something makes it more likely to happen, making planning for end-of-life preferences problematic [77]. Using ethnography and interviews, Wang *et al*. found that within the Chinese community in Australia, community stakeholders constructed new sociocultural ACP discourses that brought together both Australian and traditional Chinese values, e.g. finding ways to discuss ACP while avoiding using the word death [79].

Within current literature, there are implicit assumptions about the superiority of some narratives over others. For example, clinical guidance advising that patients should be helped to reach ‘existential maturity’, where death is seen as a ‘nontraumatic outcome’, risks implying that patients who see death as traumatic are less mature [8]. Identifying the culturally-specific nature of the narratives that shape ACP practice allows clinicians to question implicit assumptions that some types of narrative are superior to others. This is a step towards cultural humility, which begins with ‘recognising that professional norms are only one worldview’, and might help to address disparities in the quality of palliative care received by people from minoritised ethnic backgrounds [21].

As well as being culturally specific, agency and redemption narratives may have more relevance for some diseases and dying trajectories than others. Based on the analysis of a best-selling fiction book about teenagers dying from cancer, Kirkman *et al*. suggested that there are themes that are specific to adolescent end-of-life narratives, such as milestones of emerging adulthood occurring in the context of limited life expectancy. The authors argue that recognising these may help ‘adolescents to organise their thoughts and make sense of an abridged life journey’ [80].

Agency and redemption narratives may also be less relevant when there is greater uncertainty over the disease trajectory, as is often the case in frailty and advanced multimorbidity [81, 82], possibly contributing to lower rates of ACP in this population [83]. Qualitative interview research has found that many individuals with frailty feel ambivalent about ACP, not wanting to ‘waste time’ planning for an uncertain future [84], or preferring to leave decisions to others [84, 85]. This suggests the cultural narrative of control may have less relevance for this patient group, who instead prioritise living well day-to-day [84].

Further research is needed into the narratives that underpin ACP and the extent to which these are helpful for patients, particularly attending to heterogeneous cultural and socioeconomic backgrounds, illness types and life stages and the intersectionality between these factors. Research is also needed into how the narratives that underpin ACP interact with other cultural narratives, such as those about ageing and illness.

## The role of clinicians in contributing to patient narratives through ACP

We have outlined the argument that ACP can be seen as a way of helping patients to tell personal narratives that align with particular cultural narratives. We have highlighted that this can be problematic because these narratives have less relevance for some populations. Next, we turn to the role of clinicians in this process. When, as part of ACP, clinicians share information with a patient about their illness and prognosis, they can contribute to how that person thinks about and makes sense of their illness. In this way, the clinician affects the patient’s life narrative (as has been described in the context of narrative medicine more broadly—see e.g. Kalitzkus and Matthiessen and Brody [86, 87]). The clinician’s role may range from helping the patient to make sense of their illness, through to helping patients to approach dying in a particular way with, e.g. a sense of control or redemption.

We found that the role of clinicians in contributing to patient life narratives through ACP was rarely explicitly acknowledged in the literature and has never been studied empirically. Some authors view patient narratives as something to be uncovered by the clinician during ACP and do not acknowledge any role of the clinician in shaping this story [42, 51]. Where the influence of clinicians is acknowledged, views differ on what the role of clinicians should be. Some argue clinicians should shape the type of narrative their patient tells. For example, drawing on the findings of an interdisciplinary conceptual analysis, Lanocha *et al*. proposed that during ACP clinicians should ‘coconstruct the content of a patient’s narrative’ with the aim of strengthening narrative identity. They argue that this is ‘ethically preferable’ as it avoids ‘unbridled patient autonomy on the one side and clinician paternalism on the other’ [41]. Similarly, writing in a commentary on narratives and palliative care, Stanley and Hurst describe that physicians might be able to co-create a patient’s story, thereby helping the patient to make meaning at the end of life [52]. A NEJM clinical practice guideline on serious illness conversations also implies a role for clinicians in shaping their patients’ narratives, stating ‘clinicians should be able to help cultivate prognostic awareness and existential maturation’, where death is seen as a nontraumatic outcome [8].

In contrast, discussing narrative palliative care and not specifically ACP, DasGupta *et al*. problematised the facilitation by clinicians of particular stories. In a book chapter, they highlight ‘the tension between the dying person's authority over the story—and indeed her right to tell or not tell—and the palliative care worker's responsibility as an “expert” to facilitate the “good death story”.’ [88] Romanoff and Thompson argue in a commentary article that clinicians listening to stories can help those nearing the end of life to find meaning, but emphasise that there is ‘nothing that needs to be “fixed”. It is the intersubjective telling of the story in the presence of an empathic witness that fosters healing' [89].

There is a need for empirical data to understand what role clinicians currently play in co-creating patient life narratives through ACP, patients’ views on narrative co-creation and the effect on outcomes for individuals from a plurality of cultural, socioeconomic and clinical backgrounds. In the interim, we hope that this review will contribute to cultural humility by raising awareness among clinicians that they may be co-creating their patients’ life narratives through ACP, and possibly drawing on culturally-situated assumptions in the process.

Box 3 Questions for future research.

1. With respect to patients from diverse cultural, socioeconomic and clinical backgrounds, including older people living with frailty and multimorbidity:

a) Does attending to patient narratives as part of ACP improve patient-centred outcomes? If so, is this through the routes identified here, and/or through other routes?
b) What are the narrative strategies people use when talking about their preferences for end-of-life care, and what cultural narratives do they draw on?
c) To what extent and in what way do clinicians contribute to patient life narratives through ACP? If and how is this helpful to patients?

2. With respect to narratives and the care of older people more broadly:

a) What narrative approaches do clinicians use in practice when caring for older people living with frailty and multimorbidity? Do these approaches improve patient-centred outcomes, including maintaining a sense of self?
b) How do narrative approaches to ACP complement narrative approaches to older age?

## Conclusion

ACP is intertwined with narratives, yet they are often invisible: unacknowledged and not attended to explicitly in research or practice guidelines. We have sought to make visible and then unpack this black box by reviewing and synthesising the evidence from a range of literatures regarding patient and cultural narratives in ACP. We identify that clinicians paying attention to patient narratives is a route to achieving many goals of ACP, including preserving patient autonomy, strengthening relationships and preparing for the end of life. These insights into how ACP achieves its goals may improve the efficacy and effectiveness of ACP interventions by ensuring key aspects are not lost in implementation, and warrants further empirical evaluation.

We describe how ACP may help a patient to maintain their sense of self, which is important for well-being but has not previously been widely acknowledged as a potential goal of ACP. This is relevant for individuals facing serious illness and frailty, both of which can disrupt someone’s sense of self. Clinicians should be aware that ACP may affect a patient’s narrative identity, even if this is not an explicit goal, so they can choose to adjust their practice accordingly. Further research is needed to evaluate the impact of attending to patient narratives on outcomes and to explore the narrative strategies people use when speaking about their preferences for the end of life, as this may help clinicians to initiate and navigate difficult ACP conversations.

We demonstrate that ACP can be seen as a way of helping patients to tell personal narratives that align with particular cultural narratives. We highlight that this can be problematic because these cultural narratives have varying salience for different populations. We hope that illuminating the culturally-situated nature of these narratives will allow clinicians to question implicit assumptions, thereby improving cultural humility and ultimately helping to address disparities in the quality of palliative care received by people from minoritised ethnic backgrounds.

Overall, we suggest that increasing the attention and value placed on patients’ narratives during ACP, accompanied by greater cultural humility, might help to improve outcomes for patients and families.

## Supplementary Material

aa-26-0724-File002_afag191

aa-26-0724-File003_afag191
