# Supplementary material for: New horizons in advance care planning: narratives, identity and cultural humility
Source: Age Ageing. 2026 Jul 1;55(6):afag191. doi: 10.1093/ageing/afag191 (PMC13318847; doi:10.1093/ageing/afag191)
Supplement: aa-26-0724-File002_afag191 [file aa-26-0724-file002_afag191.docx]

**New horizons in advance care planning: narratives, identity, and cultural humility**

**Appendix 1**

**Review methods – further details**

For the systematic review, Medline via OVID, and Scopus were searched from inception to 12 March 2025. The search strategy was developed in collaboration with an experienced medical librarian (IK): Medline was selected for its coverage of the clinical literature and Scopus for its multidisciplinary approach, incorporating clinical, psychological and social science literature. The search included terms (freetext and MeSH where available) for advance care plan OR living will OR advance directive, combined using AND with terms for narrative OR narration. Titles and abstracts were screened by SH. All articles included for full text screening were read by two reviewers (SH,LP) who independently decided whether they met inclusion criteria. Disagreements were discussed to reach a consensus. References of included articles were searched.

**Inclusion and exclusion criteria**

**Inclusion**

Advance care planning, future care planning, advance directives, living wills or refers to this conceptually

AND

Narratives or stories or narrative medicine

**Exclusion**

ACP in general and not specifically about narratives in ACP

“Narrative reviews” not otherwise about narratives

Narrative analysis, or methods, but not about narratives in terms of content or focus

Personal narratives (as a genre) – this style of article is a description of personal experience rather than a research article, a bioethical essay or a commentary.
