# Supplementary material for: New horizons in advance care planning: narratives, identity and cultural humility
Source: Age Ageing. 2026 Jul 1;55(6):afag191. doi: 10.1093/ageing/afag191 (PMC13318847; doi:10.1093/ageing/afag191)
Supplement: aa-26-0724-File003_afag191 [file aa-26-0724-file003_afag191.pdf]

New horizons in advance care planning: narratives, identity, and cultural humility

Appendix 2: PRISMA 2020 flow diagram for new systematic reviews which included searches of databases, registers and other sources

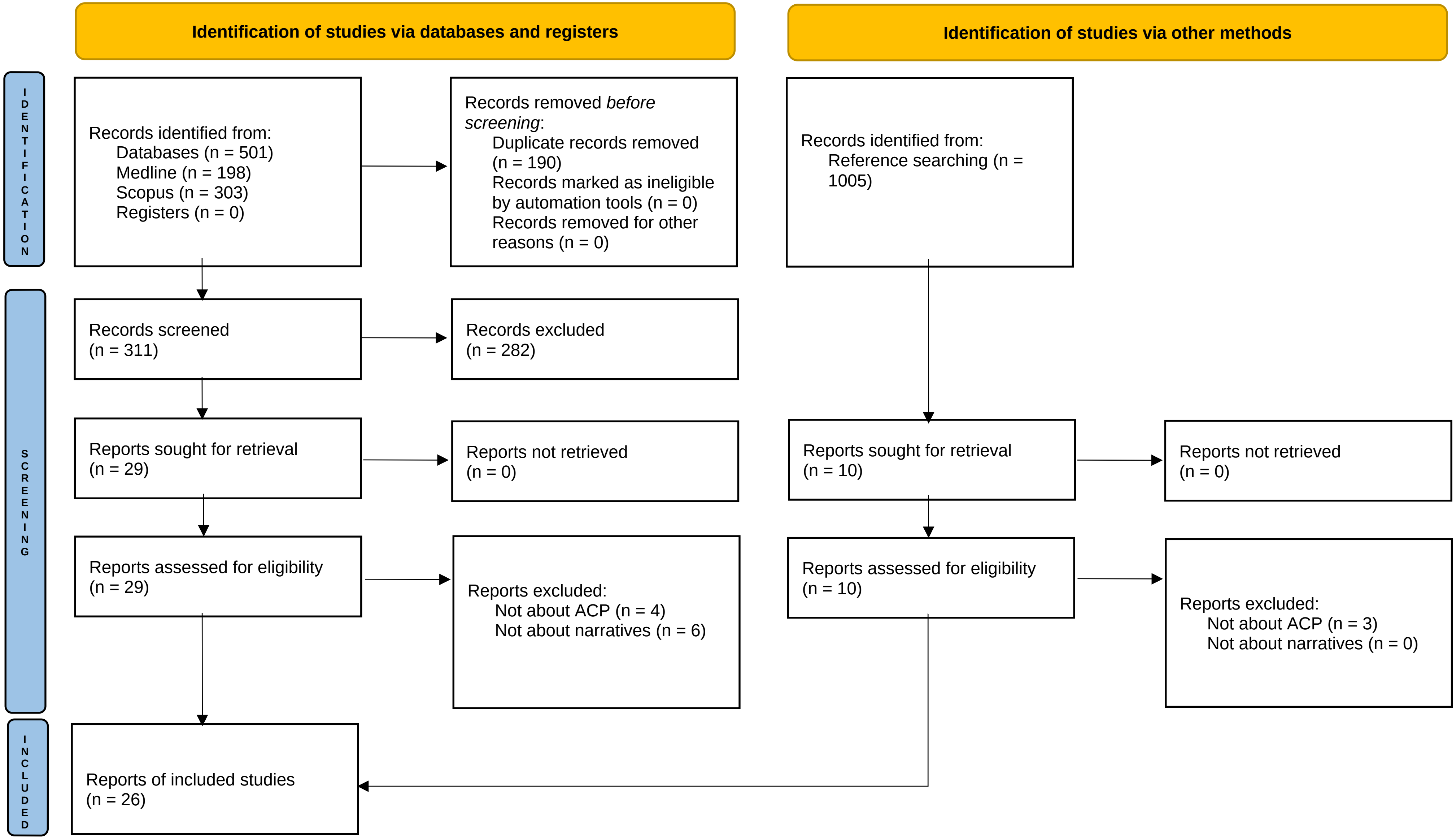

Source: Page MJ, et al. BMJ 2021;372:n71. doi: 10.1136/bmj.n71.

This work is licensed under CC BY 4.0. To view a copy of this license, visit https://creativecommons.org/licenses/by/4.0/
